# Impact of a lactobacilli-containing gel on vulvovaginal candidosis and the vaginal microbiome

**DOI:** 10.1038/s41598-020-64705-x

**Published:** 2020-05-14

**Authors:** Eline F. M. Oerlemans, Gert Bellen, Ingmar Claes, Tim Henkens, Camille Nina Allonsius, Stijn Wittouck, Marianne F. L. van den Broek, Sander Wuyts, Filip Kiekens, Gilbert G. G. Donders, Sarah Lebeer

**Affiliations:** 1 University of Antwerp, Department of Bioscience Engineering, Research Group Environmental Ecology and Applied Microbiology, Antwerp, Belgium; 2 Femicare, Clinical research for women, Tienen, Belgium; 3 YUN NV, Aartselaar, Belgium; 4 University of Antwerp, department of Pharmaceutical, biomedical and veterinary sciences, laboratory of Pharmaceutical Technology and Biopharmacy, Antwerp, Belgium; 5 Antwerp University Hospital, department of Gynaecology and Obstetrics, Antwerp, Belgium

**Keywords:** Clinical microbiology, Microbiome

## Abstract

Vulvovaginal candidosis (VVC) is a common condition with severe symptoms and high recurrence rates. Probiotic lactobacilli are explored as alternatives to azole treatments. Although the vaginal microbiota is generally not depleted in lactobacilli during VVC, studies indicate that the functionality and antimicrobial activity of the lactobacilli is impaired. We selected three strains from the *Lactobacillus* genus complex (*L. rhamnosus* GG, *L. pentosus* KCA1 and *L. plantarum* WCFS1) based on *in vitro* evaluation and formulated them in a gel for vaginal application. This gel was evaluated in 20 patients suffering from acute VVC, who were followed for four weeks including a 10-day treatment period. The microbiome was assessed through *16S rRNA* (bacteria) and internal transcribed spacer (ITS; fungi) amplicon sequencing, supplemented with quantitative PCR, culture and microscopy for *Candida* evaluation. 45% of women did not require rescue medication (3×200 mg fluconazole), implying an improvement of their symptoms. These women showed similar end concentrations of fungi as women treated with fluconazole. Moreover, fluconazole appeared to reduce numbers of endogenous lactobacilli. Our study points towards important aspects for future selection of lactobacilli for probiotic use in VVC and the need to investigate possible negative influences of azoles on the vaginal bacterial community.

## Introduction

During their lifetime, 75% of women will experience at least one episode of vulvovaginal infection by *Candida* species, i.e. vulvovaginal candidosis (VVC). Patients exhibit various symptoms such as vaginal itching or soreness, dyspareunia, abnormal vaginal discharge, redness, swelling and thinning of the vaginal wall^1^. While azole treatment is often fast and effective in eradicating VVC, azole resistance in *Candida* is increasingly detected in recurrent infections^1^. Since azoles are currently available as over-the-counter drug, women who self-diagnose and mistreat could aggravate these issues, stressing the need for alternative treatments and/or complementary aids.

Several species and strains from the *Lactobacillus* genus complex (LGC; recent taxonomic reclassification has divided the former genus of *Lactobacillus*, now referred to as the LGC, into several new genera^2^), also referred to as lactobacilli, are known for their health-promoting effects. These health effects are most often attributed to their anti-pathogenic and immunomodulatory properties^3^. Supplementation of lactobacilli is hypothesized to have great potential for restoring vaginal health, as the vaginal microbial community of healthy women is most often dominated by one of four *Lactobacillus* species: *L. crispatus*, *L. iners*, *L. gasseri* or *L. jensenii*^4^. However, a protective role for these endogenous lactobacilli against *Candida* infections is unclear. Previous studies have indicated that these *LGC* species are generally present in the vaginal niche throughout an episode of VVC and thus appear not to offer sufficient protection to prevent or reduce the growth and/or pathogenicity of *Candida*^5–7^. On the other hand, there are indications that while lactobacilli remain present, they appear to be reduced in abundance^8^, which may impact their efficacy to control *Candida* virulence. Some protective effects of supplementation of specific strains of the LGC and formulations in clinical settings of VVC have indeed been reported^9–13^, but not all studies were successful^14^. Here, we therefore rationalized that LGC supplementation could have benefits for the prevention and treatment of VVC, provided a selection of strains with strong anti-*Candida* properties is performed.

First we explored in detail the *in vitro* inhibitory effects of a selection of LGC strains against growth and pathogenic properties (adhesion to vaginal cells and hyphae formation) of *Candida albicans*. It is increasingly appreciated that a combination of targeting both the inhibition of pathogenic growth *and* virulence factor expression has superior efficacy in eliminating pathogens^15^. One of the simplest, yet very effective ways that lactobacilli inhibit the growth of competing bacteria is through the production of lactic acid. It is likely various *C. albicans* strains exhibit various levels of acid resistance but lactic acid has been shown to inhibit *Candida albicans*^16^. As lactobacilli exhibit various levels of lactic acid production^17^, we rationalized here that the selected lactobacilli should have a strong intrinsic capacity to produce lactic acid, at least in the optimal growth conditions. Because of the indication that endogenous vagina-specific lactobacilli do not always sufficiently protect against *Candida*, we focused on the selection of habitat-flexible lactobacilli, mainly from the *L. plantarum* and *L. casei* group. Although most LGC species of the strains tested here have “qualified presumption of safety”^18^ status when applied orally in food and feed, such guidelines do not yet exist for vaginal topical applications of lactobacilli. To reduce safety risks also other important factors were taken into account, such as genome availability^19–21^, previous knowledge of their human host interaction capacity (primarily in the gut)^22,23^, their robustness and growth capacity^19,21,23^, and previous reports on their safety in humans after oral^24–26^, nasal^27^ and vaginal^28^ high-dose application. We selected three strains and formulated them into an innovative silicon-based gel, which was designed specifically for application in the vagina and maintained a high viability of the lactobacilli. This gel was supplied to patients with acute VVC for 10 days. We examined the vaginal microbiome over four weeks: before, during, and after the use of the probiotic gel and compared the group requiring rescue medication (RM; 3 × 200 mg fluconazole) with the group not requesting this antifungal treatment.

## Results

### *In vitro* selection of LGC strains for anti-*Candida* effects

**Figure 1:**
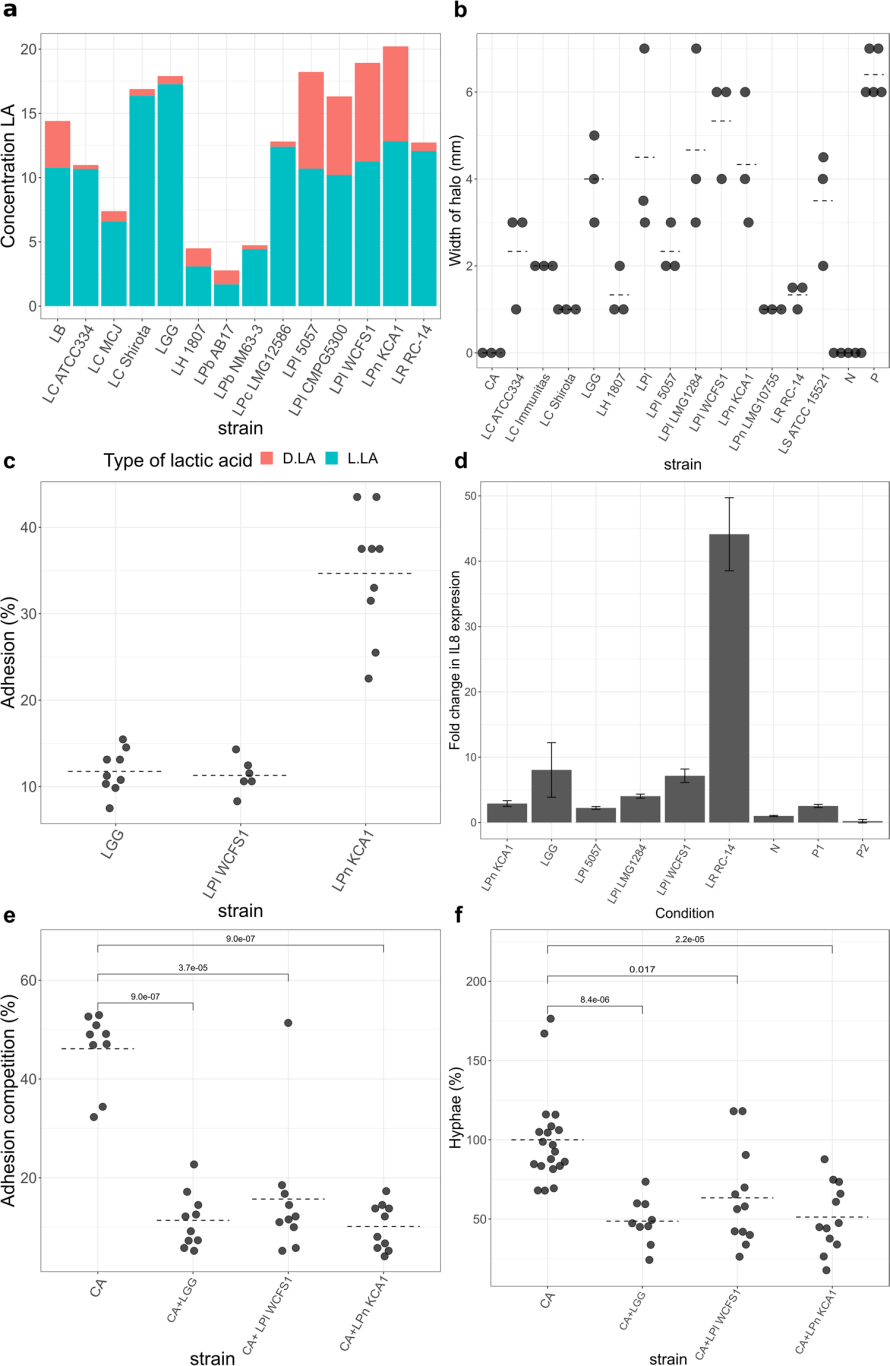
Anti-*Candida* activity of LGC strains (**a**) Production of D- and L-lactic acid as important antimicrobial compound secreted by the tested LGC strains (**b**) Width of halo of inhibition zone (radius of spot subtracted from radius of inhibition zone) around LGC strains in competition with *C. albicans* in the spot assay. Halos around the LGC colonies indicate inhibition by the strain against *C. albicans*. (**c**) Adhesion percentage of LGC strains to VK2/E6E7 cells, a cell line of vaginal keratinocytes. (**d**) Fold increase in interleukin-8 expression by co-incubation of the LGC strains (10^7^ cells/ml) with VK2/E6E7 cells. The regular growth medium without any added microorganisms was used as a negative control. (**e**) Adhesion of *C. albicans* to VK2/E6E7 cells either alone or in co-incubation with lactobacilli as percentage of adherent cells to total applied cells. (**f**) Formation of hyphae by *C. albicans* as ratio of number of yeast cells showing hyphae to total cells, normalized to the negative control (=1). LA: lactic acid, N: negative control, P: positive control, CA: *Candida albicans*, LB: *Lactobacillus bulgaricus*, LC: *L. casei*, LGG: *L. rhamnosus* GG, LH: *L. helveticus*, LPb, *L. parabuchneri*, LPl: *L. plantarum*, LPn: *L. pentosus*, LR: *L. reuteri*, LS: *L. sakei*.

We screened our selection of LGC strains for direct growth inhibition against *Candida* (Fig. 1a,b), adhesion to the epithelium (Fig. 1c), absence of induction of an inflammatory marker (Fig. 1D) and inhibition of adhesion to and invasion of the host (Fig. 1e,f).

Concentrations of produced lactic acid (Fig. 1a) ranged from 2.78 g/L (*L. parabuchneri* AB17) to 20.22 g/L (*L. pentosus* KCA1). *L. rhamnosus* GG produced the highest amount of L-lactic acid (17.27 g/L) and *L. plantarum* WCFS1 produced the highest amount of D-lactic acid (7.68 g/L). The latter also performed the best in the *C. albicans* growth inhibition assays. In the spot assay, it yielded the largest inhibition zones (5.3 mm; Fig. 1b) and its cell free supernatant reduced the optical density at stationary planktonic growth of *C. albicans* by 43.5% and delayed the lag phase by approximately 5 hours (Supplementary Fig. S1).

The lactobacilli with the strongest antimicrobial effects showed adhesive capacities of 11.3% (*L. plantarum* WCFS1), 11.8% (*L. rhamnosus* GG) and 34.7% (*L. pentosus* KCA1) (Fig. 1c) to vaginal keratinocyte cell line (VK2/E6E7), used as model for the vaginal epithelium. Co-incubation of these bacteria and keratinocytes did not provoke an elevation of interleukin 8 expression, neither did two other *L. plantarum* strains, but *L. reuteri* RC-14 did cause some upregulation of interleukin 8 (Fig. 1d). When applying the strains simultaneously with *C. albicans*, the strains were able to reduce adhesion of *C. albicans*, e.g. *L. pentosus* KCA1 by 78.0% (Fig. 1d). Finally, we compared the capacity of the lactobacilli to inhibit the formation of hyphae, a key virulence factor of *C. albicans* that enables invasion of host tissue. *L. pentosus* KCA1, *L. plantarum* WCFS1 and *L. rhamnosus* GG all showed strong inhibitory effects. The latter exhibited the strongest effect (reducing hyphal formation by 51.3%; Fig. 1e).

### Formulation of lactobacilli in silicone gel to preserve viability and promote topical applications

Based on the above screening, three LGC strains were selected i.e. *L. plantarum* WCFS1, *L. pentosus* KCA1 and *L. rhamnosus* GG. These strains were formulated in an innovative silicone gel at a dose of 10^9^–10^10^ CFU/g gel, in a 1:1:1 weight ratio, as described in materials and methods. The vaginal gel was developed to ensure maximal exposition of living bacteria to the vaginal wall through spreading after intravaginal application and maintain a high viability. Plate count viability assays confirmed that the lactobacilli remained viable over multiple months of storage at 5 °C and 25 °C (Supplementary Fig. S2). For storage at 5 °C, no reduction could be observed in viability over the two-year period. The concentration of viable lactobacilli did decline during storage at 25 °C, showing roughly a ten-fold reduction after 6 months of storage.

### Proof-of-concept study in patients with VVC

**Figure 2:**
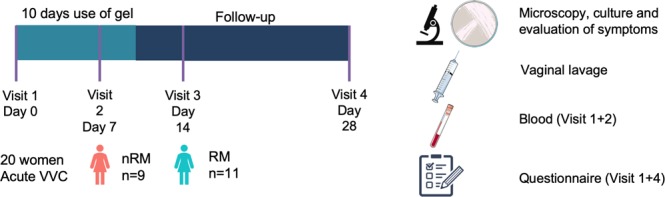
Overview of the proof-of-concept study. Twenty women with acute VVC were recruited for the proof-of-concept study where they were asked to use a vaginal gel containing lactobacilli once daily before bedtime over the course of 10 days. Patients were asked to return for evaluation of symptoms and VVC diagnosis (through microscopy and culture) one, two and four weeks after the intake visit. At each visit vaginal lavage fluid was collected for *16 S rRNA* and ITS sequencing and qPCR. For evaluation of the gel’s safety, a blood sample was collected at the first two visits. Data on medical history and patient satisfaction was collected through questionnaires at the intake visit and study termination (visit 4). In this figure, images from Servier Medical Art (http://smart.servier.com/) were used unchanged, licensed under a Creative Commons Attribution 3.0 Unported License (https://creativecommons.org/licenses/by/3.0/).

Subsequently, we evaluated whether *L. plantarum* WCFS1, *L. pentosus* KCA1 and *L. rhamnosus* GG formulated in the gel were able to modulate the vaginal microbiome over a four-week period in 20 patients suffering from acute VVC (Fig. 2). The participants were asked to administer 2.5 ml of gel by use of an applicator in recumbent position at bedtime. This corresponded to 2.5.10^9^–10^10^ CFU of bacteria per application or 2.5-250 times the amount of bacteria in 1 ml of vaginal discharge^29^. Because of the acute phase of the disease, patients had access to RM (3 × 200 mg of fluconazole), as requested by the ethical committee in this phase of the study. Because fluconazole is available as standard care, this study design with a rescue arm was preferred over a placebo-controlled trial. No safety issues of the vaginal LGC gel were reported. Of the twenty women included in the study, nine women (45%) did not need the RM. Eleven women used RM (3 × 200 mg of fluconazole), starting after on average 9.8 days (range: 5–24 days).

**Figure 3:**
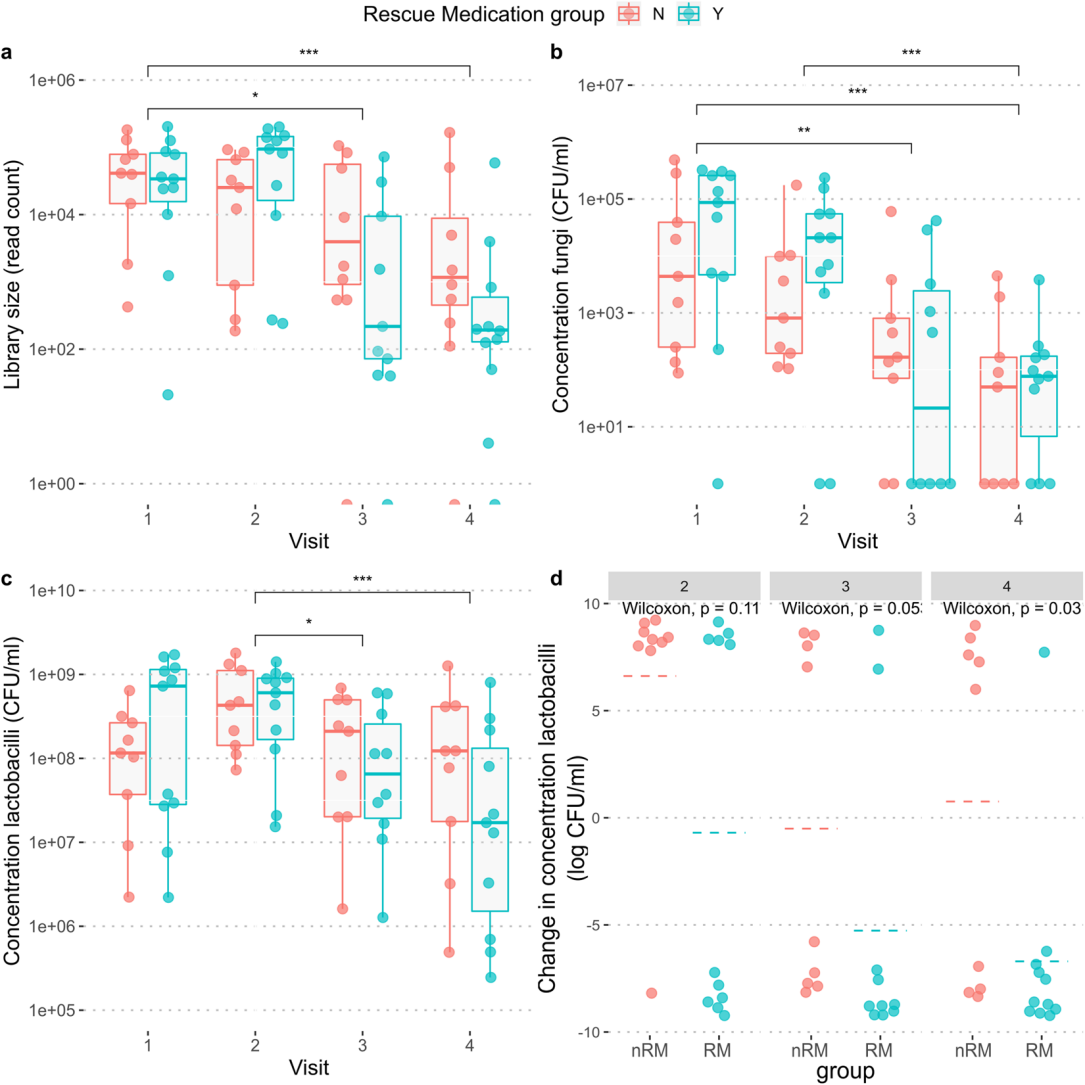
Estimations of bacterial and fungal concentrations over the course of the study: sequencing depth for ITS sequencing (**a**), estimated absolute abundances of fungi (**b**) and lactobacilli (**c**) and change in LGC concentration as compared to the first visit (**d**). Samples are divided by visit and colored by group: women requiring RM are indicated as a blue dot and women who only used the probiotic gel are colored red. Significant differences were tested with pairwise Wilcoxon tests and significance levels are indicated when p < 0.05. *p < 0.05, **p < 0.01, ***p < 0.005. RM: RM group, nRM: non-RM group.

The microbiome was investigated at four time points (at intake= day 0, day 7, day 14 and day 28). 36 out of 80 samples (20 samples over 4 timepoints) showed a relatively low sequencing depth (fewer than 2000 reads over two cumulated technical repeats; Fig. 3a), particularly samples of the third and fourth visits. The qPCR results showed that in general the estimated absolute fungal abundances were significantly lower for these visits (visit 3 mean=7.4.10^3^ CFU/ml and visit 4 mean=5.7.10^2^ CFU/ml) as compared to visit 1 (mean = 1.1.10^5^ CFU/ml, p = 0.005 and p = 0.0002 respectively, visit 2 mean = 3.8.10^4^ CFU/ml, visit 2 vs. 4 p = 0.0009; Fig. 3b). As for the estimated absolute abundances of lactobacilli in the samples, the total concentrations of combined endogenous and applied lactobacilli did not increase significantly from the first visit (mean visit 1 = 4.5.10^8^ CFU/ml, mean visit 2 = 6.1.10^8^ CFU/ml). This despite that patients had administered an estimated number of 2.5.10^9^–10^10^ CFU of lactobacilli the night before the second visit. The total concentration even slightly dropped after the second visit, yielding significantly lower concentrations of lactobacilli at visit 3 and visit 4 (mean visit 3 = 2.1.10^8^ CFU/ml, mean visit 4 = 1.9.10^8^ CFU/ml; visit 2 vs. 3 p = 0.034, visit 2 vs. 4 p = 0.004, Fig. 3c). Of note, when we stratified the samples in women who did or did not use RM, the following trend was observed: the women who used RM started off with a higher load of fungi and lactobacilli and ended with on average with similar fungal concentrations and lower concentrations of lactobacilli as compared to the women who did not use RM (Fig. 3). These differences were not statistically significant at each time point, because of large variations within the two groups and a lack of statistical power (11 versus 9 subjects; p-values ranging from 0.22 to 1, Supplementary Fig. S3). However, when the concentration difference between the first and the last visit was evaluated, a larger decrease in the concentration of lactobacilli was observed in the RM group as compared to the group without RM that was statistically significant (p = 0.03). While we observed a decline in LGC concentration for the RM group, the LGC concentration in the non-RM remained relatively stable (Fig. 3d). This difference between the RM and non-RM group was not observed for fungal concentrations (Supplementary Fig. S4).

### Analysis of fungal community in VVC

**Figure 4:**
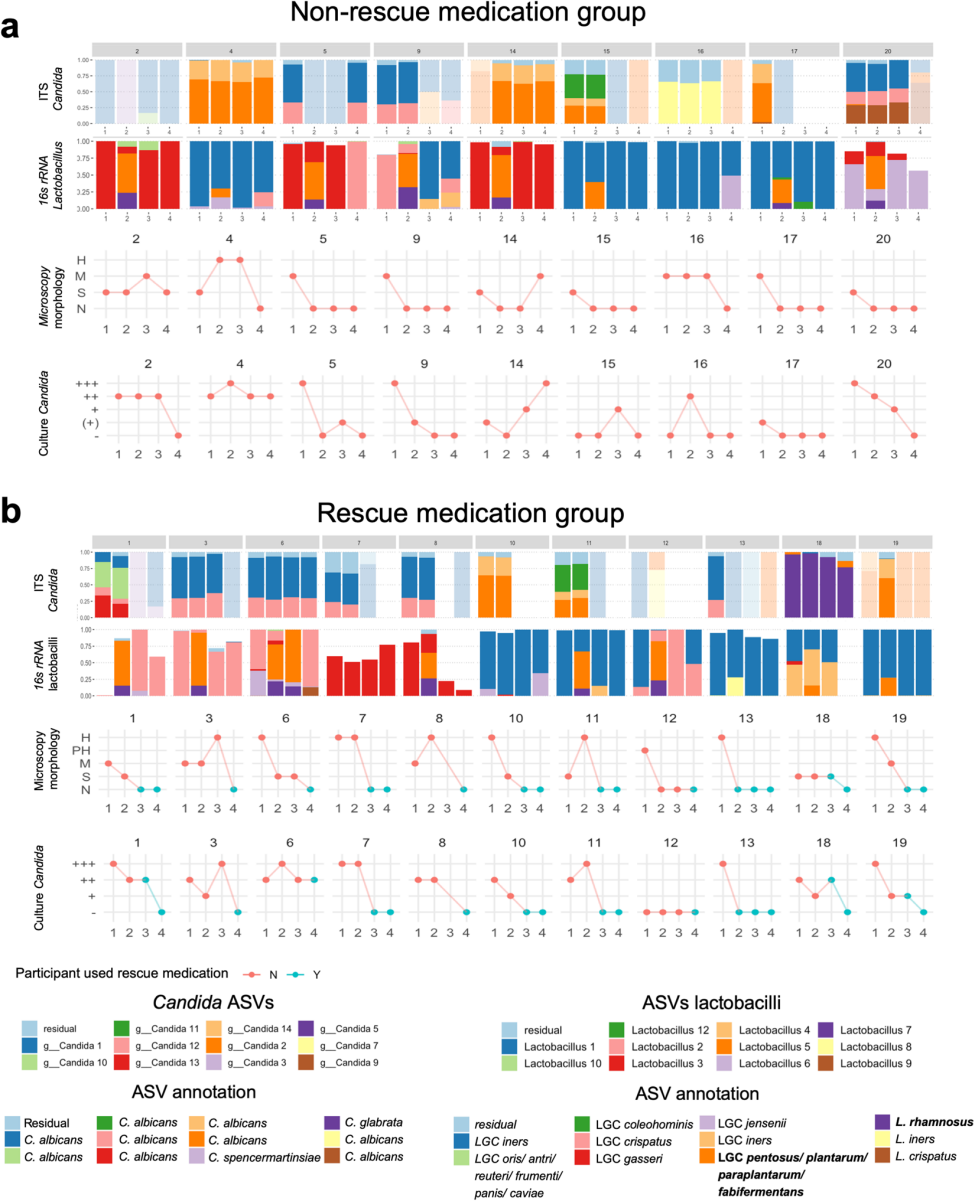
Evaluation of the microbiome of the samples through sequencing, microscopy and culture. Results of ITS sequencing focused on *Candida* (top row), *16 S rRNA* sequencing focused on LGC (2^nd^ row), microscopy for *Candida* morphology (3^rd^ row) and culture of *Candida* (bottom row) for women who did not use RM (A) and women who did use RM (B) are indicated. Samples are ordered by patient and subsequently by visit. ASVs were classified by EZBioCloud^30^. Two ASVs that likely result from the supplemented lactobacilli are indicated in bold. Abbreviations for morphology in microscopy: N: no *Candida* observed, S: sporae, M: mixed, PH: pseudohyphae, H: hyphae.

We subsequently analyzed the taxonomic composition of the fungal community (Fig. 4). The samples with sufficiently high read counts (>2000 reads) were all almost completely dominated by *Candida* amplicon sequence variants (ASVs) (mean relative abundance 99.9%; range: 98.8%–100%). In total 24 *Candida* ASVs were found in the dataset, of which the eleven most abundant were plotted in Fig. 4 and classified to a sub-genus level by EZ BioCloud^30^. Nine of these eleven ASVs were identified as *Candida albicans*, one as *Candida glabrata* (*Candida* 5) and one as *Candida spencermartinsiae* (*Candida* 3). We found that *Candida* 1 and *Candida* 12 on the one hand and *Candida* 2 and *Candida* 14 on the other hand always occurred together, and usually in similar ratios. Since these four ASVs were also determined as *C. albicans*, this indicates that these sequences might actually be derived of the same organism. Based on our classification, 17 of the 20 women who participated in the study showed infection by *Candida albicans*, while in one woman the infection was caused by *Candida glabrata* (Fig. 4a,b) and in two other we did not obtain sufficient sequencing depth. This concurred with the culture data, commonly used for VVC diagnosis. Based on these culture data, we could also identify *Candida krusei* in one of these two women.

### Analysis of LGC community in VVC

We also investigated the bacterial community present in the samples. LGC was the most abundant bacterial genus, both during and after resolving VVC, accounting for more than 90% of reads in the large majority of the samples and six of the 11 most abundant ASVs belonging to this genus (Fig. 4, Supplementary Fig. S5). The other ASVs belong to the genera *Gardnerella, Atopobium, Prevotella, Aerococcus* and *Streptococcus*. Seven of the most dominant LGC ASVs matched with the four species typically observed in the vaginal microbiota, more precisely *L. iners* (LGC 1, 4 and 8)*, L. crispatus (*LGC 2 and 9*), L. gasseri* (LGC 3) and *L. jensenii* (LGC 6). Two other ASVs were classified as *L. pentosus/ plantarum/paraplantarum/ fabifermentans* (LGC 5) and as *L. rhamnosus (*LGC 7). This first ASV likely corresponds to *L. pentosus* KCA1, *L. plantarum* WCFS1 or both, which were supplemented in the gel, as this ASV was only found at visit 2 (16/20 samples, during treatment with the gel) and visit 3 (1/20 samples). Similarly, LGC 7, found in 12 of the 20 visit 2 samples and one visit 3 sample, likely corresponded to *L. rhamnosus* GG, also supplemented in the gel. This ASV showed a lower relative abundance than *L. plantarum/pentosus* (mean relative abundance at visit 2: 11.0% versus 37.9% of LGC 5). Finally, LGC 10 and 12 were classified as *L. oris/antri/reuteri/frumenti/panis/caviae* and *L. coleohominis*.

Specific taxa of lactobacilli could not be linked to RM use but the bacterial community of the samples from women who used RM did seem to show somewhat higher relative abundance of non-LGC genera as compared to the samples derived from women who did not use RM (p = 0.057). In most women (15/19) the lactobacillus that dominated the bacterial community before treatment (visit 1) was also dominant at the end of the study, after the treatment (visit 4), indicating the applied lactobacilli had a temporary effect on the microbiome (Supplemental Fig. S7). We did observe shifts in relative abundances of the lactobacilli. Two women ended the study with a different dominant *Lactobacillus* as at the study onset and two women had bacterial community profiles not dominated by *Lactobacillus* at visit 1 or 4. One visit 4 sample could not be analyzed because of insufficient sequencing depth.

## Discussion

The high recurrence of VVC and limited treatment options require the development of alternative therapies. Although probiotics, mainly LGC species, have been suggested as a possible therapy, they are not frequently applied. Here, three LGC strains were selected for further evaluation in patients based on *in vitro* anti-*Candida* effects. We showed their ability to inhibit *Candida albicans in vitro* and formulated them in a gel, which was tested in a proof-of-concept study aimed at investigating their influence on the vaginal microbiome and highlighting relevant characteristics for future improvements for probiotic strategies against VVC.

We found that specific LGC strains can be selected to compete with *Candida* from *in vitro* findings and inhibit *Candida* sufficiently *in vivo* under certain conditions, as indicated by the reduction in fungal concentrations in the RM and the non-RM group to similar levels. Not all women who participated were sufficiently helped with the probiotic gel (as can be seen in the clinical composite scores of visit 2, Supplemental Fig. S6), but these women likely suffered from more severe infections. In this group we found higher concentrations of fungi at study onset, higher occurrence of hyphae and a higher number of previous infections. However, although our test probiotic formulation was not successful for all treated patients, it might still be useful as adjuvant treatment for severe cases. Previous studies have reported successes in using probiotics for VVC treatment (in combination with azole treatment), but these were mostly aimed at reducing the rate of recurrence and did not test the lactobacilli as stand-alone treatments for acute pathology^10–13,31^. For the milder cases, it could be a stand-alone treatment. The latter is suggested by the fact that in women without need for RM, the symptoms and lab findings did improve rapidly (within a few days) when using the gel and continued to improve during the weeks following treatment. The product did not show notable side effects and was even felt as an agreeable gel to use, leading to the willingness to use it again in all women who did not use RM, and surprisingly even in half of the women who eventually did need RM. This suggests that there is high acceptance to use this gel, even if it did not solve all symptoms in all patients. Lastly, women using RM ended with lower lactobacilli in the qPCR data as compared to women who did not use RM (reversed to the situation of at the intake visit). Thus, our data suggest that the use of the RM targeting the fungal community might have a negative effect on the present bacteria, diminishing numbers of lactobacilli, which are the hallmark of a healthy vaginal microbiome. This finding suggests more research is necessary concerning the vaginal microbiome also after clearing an episode of VVC. The decline in LGC concentrations should be investigated in larger cohorts that preferably also evaluate if this is linked to RM use, or possibly disease severity, specific symptoms or microbiota members and ultimately recurrence rates. Nonetheless, whatever the underlying reason may be, there might be a role for probiotics in preventing or restoring such a drop in beneficial lactobacilli. In addition, the data obtained here also form a starting point for future improvements of similar probiotic treatments for VVC, in hope to obtain even better cure rates. First, future *in vitro* screening should include experiments predicting the *in vivo* survival and adaptability to the vaginal environment, given that our strong antimicrobial *L. rhamnosus* GG strain did not persist well in the vagina. For this reason, closely related strains that share (some) of their properties with *L. rhamnosus* GG, but which were isolated from the vagina might be interesting alternative probiotics. Secondly, based on our ASV analysis we expect that there are (at least) two main types of *C. albicans* that occur often in VVC. Isolation and characterisation of these two types of *C. albicans* should confirm their pathogenicity. If confirmed, selection of new probiotic targets active against these two pathogenic forms would definitely benefit the chances of success for future probiotic VVC studies.

## Materials and methods

### Culture of micro-organisms

An overview of the used micro-organisms can be found in Table 1. Lactobacilli were grown in de Man, Rogosa and Sharpe (MRS) medium and *Candida albicans* in yeast extract peptone dextrose (YPD) medium at 37 °C. For solid media, 5%w/v (for overlay) or 15% w/v (regular base) of microbiological agar (VWR) was supplemented to the medium.

**Table 1:** Inventory of bacterial strains used in the *in vitro* work of this chapter.

Strain	Source/reference
*Candida albicans* SC5314	Martin *et al*.^44^
*L. bulgaricus* AMB-1	Yoghurt
*L. casei* ATCC334	American Tissue Culture Collection
*L. casei* MCJ	Fermented food
*L. casei* Shirota	Commercial probiotic product
*L. helveticus* 1807	Commercial probiotic product
*L. parabuchneri* AB17	Fermented food
*L. parabuchneri* NM63-3	Fermented food
*L. paracasei* LMG12586	Belgian Coordinated Collections of microorganisms
*L. pentosus* KCA1	Anukam *et al*.^21^
*L. pentosus* LMG10755	Belgian Coordinated Collections of microorganisms
*L. plantarum* 5057	Danielsen, 2002^45^
*L. plantarum* LMG1284	Belgian Coordinated Collections of microorganisms
*L. plantarum* CMPG5300	Malik *et al*.^46^
*L. plantarum* WCFS1	Kleerebezem *et al*.^20^
*L. reuteri* RC-14	Chan *et al*. 1984 & 1985, Reid & Bruce 2001, Reid & Reid 1999^28,47–49^/ ATCC
*L. rhamnosus* GG	Kankainen *et al*.^19^

### Lactic acid production

To evaluate lactic acid production, lactobacilli were grown to stationary phase, after which the cultures were centrifuged (2000 g, 10 minutes) and the supernatant passed through a sterile 0.2 µm cellulose filter (VWR). Levels of lactic acid in the cell-free supernatant (CFS) were tested with the D-Lactic/L-lactic acid kit (r-biopharm), according manufacturer’s instructions.

### Spot assay for growth inhibition and inhibition of hyphae formation in *C. albicans* by lactobacilli

To evaluate the potency of the LGC strains to inhibit the growth of *Candida albicans* (inoculated at 2.10^3^ CFU/ml), a spot assay was performed as described previously^32^.

The ability of the LGC strains to inhibit hyphae formation was measured as described previously^33,34^.

### Adhesion of lactobacilli and adhesion-competition of lactobacilli and *C. albicans* to VK2/E6E7cells

The vaginal keratinocyte immortalized cell line, VK2/E6E7, was used as model for the vaginal epithelium and obtained from the Center for Molecular Plant Genetics (CMPG) in Leuven, Belgium.

The potency of lactobacilli to adhere to VK2/E6E7 cells or compete for adhesion sites with *C. albicans*, was evaluated as described previously^33–35^ and details can be found in supplementary information. Briefly, suspensions of lactobacilli (1.10^8^ for adhesion assay or 2.10^8^ CFU ml^−1^ for adhesion competition assay) and *C. albicans* (2.10^6^ CFU ml^−1^, only for adhesion competition assay) were co-incubated with VK2/E6E7 cells for 2 h, after which unattached bacteria were washed away and cells were detached. Concentrations of lactobacilli and *C. albicans* in these cell suspensions were determined through plating of serial dilutions.

### Induction of interleukin 8 expression in VK2/E6E7 cells by lactobacilli

VK2/E6E7 cells were cultured to monolayers as described previously^35^. Solutions of 10^7^ CFU/ml of the lactobacilli were prepared as described for the adhesion experiments, added to the cells and co-incubated for 1.5 h. After washing the cells twice with PBS, RNA was extracted using the RNeasy Mini kit (Qiagen), according to manufacturer’s instructions, and stored at −80 °C. RNA concentrations were estimated with NanoDrop 1000 (Thermo Scientific). 1 µg of RNA was used for cDNA synthesis using oligo-(dT) primers and ReadyScript® reverse transcriptase (Sigma Aldrich), according to the manufacturer’s instructions. qPCR for interleukin 8 and reference genes, *peptidylprolyl isomerase A (PPIA)* and *glyceraldehyde 3-phosphate dehydrogenase (GAPDH)* was performed as previously described^32^. Primer sequences can be found in Supplementary Table 3.

### Formulation of gel containing selected lactobacilli

*L. pentosus* KCA1, *L. plantarum* WCFS1 and *L. rhamnosus* GG, were selected for a proof-of-concept trial in patients. The lactobacilli-containing gel was developed in collaboration with the Belgian biotech company YUN nv (see ‘Competing interest’ statement). The selected LGC spp. were spray-dried and formulated in a silicon-based gel containing 85.5% dimethicone, 4.5% bis-vinyl dimethicone/dimethicone copolymer and 10% LGC powder mix (strains were blended in equal amounts based on weight) resulting in a colloid suspension-gel (final dosage per gram of 10^9^−10^10^ CFU of lactobacilli).

### Proof of concept study for evaluation of LGC-containing gel, ethical approval and informed consent

The effectiveness and tolerability of the gel, as well as its effect on the microbiome was evaluated in acute VVC patients, recruited from the vulvovaginitis clinic in Femicare, the Regional Hospital Tienen, and the University hospital Antwerp, Belgium. The study was performed according to the study protocol approved by the Central Ethical Committee of the University Hospital Antwerp and the local ethical committee (B300201628296 - 16/7/66; Clinicaltrails.gov identifier: NCT03975569). All methods were performed in accordance with the relevant guidelines and regulations. All participants were evaluated for exclusion and inclusion criteria (Supplementary Table S1) and were informed about the study aim and protocol through written informed consent for study participation.

After signing informed consent forms, patients were asked to administer 2.5 ml of the gel daily for 10 consecutive days. RM was provided to patients to be used as necessary: oral fluconazole 3 × 200 mg. Patients were monitored over four weeks and asked to return three times after the intake visit (day 0): at day 7 (visit 2, during treatment with gel), day 14 (visit 3) and day 28 (visit 4). At all visits, a gynaecological examination was performed, which included the scoring of symptoms, collection of a vaginal smear for pH, two vaginal swabs (for microscopy and culture) and collection of vaginal lavage samples. The following symptoms were scored as absent (0), mild (1), moderate (2) or severe (3) and cumulated for the clinical composite score: vulvovaginal itching, burning, redness, fissures and edema. Vaginal rinsing fluid was collected in a standard way^36^. Two questionnaires were collected; at the entry visit (medical history and demographics) and at the last visit (satisfaction).

### Evaluation of the microbiome of patient samples

The vaginal lavage fluids obtained were stored frozen at −80 °C until microbiome analysis, performed as described in De Boeck *et al*.^37^, with minor modifications. Briefly, DNA was extracted from the vaginal lavage samples (PowerFecal DNA extraction kit, Qiagen) and subjected to respectively 25 and 30 cycles of PCR amplification targeting the V4 region of the *16*S *rRNA* gene (for bacteria; Supplementary Table 2) and the ITS2 region (for fungi; Supplementary Table 3)^38,39^. PCR products were purified (AMPure XP PCR purification, Beckmann Coulter) and pooled equimolarly. The subsequent libraries (one bacterial and one fungal) were sequenced on separate Illumina Miseq runs (v2 chemistry, 2×250 kit, Illumina). Quality control and processing of reads was performed using the R package DADA2, version 1.6.0^40^, which included merging of reads and removal of reads with conflicting bases or chimeric sequences. Finally, ASVs were classified from the kingdom to the genus level using the EzBioCloud and UNITE databases^30,41^. A species annotation was added to each ASV by listing the species of all *16 S rRNA* sequences in the database that showed an exact match to the ASV sequence. Contaminants were identified using the approach of Jervis-Bardy *et al*.^42^, based on a p-value <0.0001. The in-house R package “tidyamplicons” (publicly available at github.com/SWittouck/tidyamplicons) was used for processing of the ASV table and annotation of metadata to samples.

### Estimation of LGC and fungal concentrations using qPCR

To supplement the microbiome data with estimations of absolute abundances of lactobacilli and yeast, the DNA samples obtained before were subjected to qPCR analysis, as described previously^43^.

## Supplementary information

Supplementary information.

## Data Availability

The obtained sequencing data and metadata is available under ENA accession number PRJEB33108.

## References

[1] Chew SY, Than LTL (2016). Vulvovaginal candidosis: Contemporary challenges and the future of prophylactic and therapeutic approaches. Mycoses.

[2] Wittouck S, Wuyts S, Meehan CJ, van Noort V, Lebeer S (2019). A Genome-Based Species Taxonomy of the Lactobacillus Genus Complex. mSystems.

[3] Lebeer, S., Vanderleyden, J. & De Keersmaecker, S. C. J. Genes and molecules of lactobacilli supporting probiotic action. Microbiol. Mol. Biol. Rev. 72, 728–64, Table of Contents (2008). 10.1128/MMBR.00017-08 PMC2593565 19052326

[4] Petrova MI, Lievens E, Malik S, Imholz N, Lebeer S (2015). Lactobacillus species as biomarkers and agents that can promote various aspects of vaginal health. Front. Physiol..

[5] Mcclelland RS (2009). A Prospective Study of Vaginal Bacterial Flora and Other Risk Factors for Vulvovaginal Candidiasis. J. Infect. Dis..

[6] Zhou X (2009). Vaginal microbiota of women with frequent vulvovaginal candidiasis. Infect. Immun..

[7] Macklaim JM, Clemente JC, Knight R, Gloor GB, Reid G (2015). Changes in vaginal microbiota following antimicrobial and probiotic therapy. Microb. Ecol. Health Dis..

[8] Liu, M. B. et al. Diverse vaginal microbiomes in reproductive-age women with vulvovaginal candidiasis. Plos One **8**, (2013). 10.1371/journal.pone.0079812 PMC3827160 24265786

[9] Ehrström S (2010). Lactic acid bacteria colonization and clinical outcome after probiotic supplementation in conventionally treated bacterial vaginosis and vulvovaginal candidiasis. Microbes Infect..

[10] Kovachev SM, Vatcheva-Dobrevska RS (2014). Local Probiotic Therapy for Vaginal Candida albicans Infections. Probiotics Antimicrob. Proteins.

[11] Pendharkar S, Brandsborg E, Hammarström L, Marcotte H, Larsson PG (2015). Vaginal colonisation by probiotic lactobacilli and clinical outcome in women conventionally treated for bacterial vaginosis and yeast infection. BMC Infect. Dis..

[12] Palacios S, Espadaler J, Fernández-Moya JM, Prieto C, Salas N (2016). Is it possible to prevent recurrent vulvovaginitis? The role of Lactobacillus plantarum I1001 (CECT7504). Eur. J. Clin. Microbiol. Infect. Dis..

[13] Martinez RCR (2009). Improved treatment of vulvovaginal candidiasis with fluconazole plus probiotic Lactobacillus rhamnosus GR-1 and Lactobacillus reuteri RC-14. Lett. Appl. Microbiol..

[14] Pirotta M (2004). Effect of Lactobacillus in preventing post-antibiotic vulvovaginal candidiasis: A randomised controlled trial. BMJ..

[15] Clatworthy AE, Pierson E, Hung DT (2007). Targeting virulence: A new paradigm for antimicrobial therapy. Nat. Chem. Biol..

[16] Tachedjian G, Aldunate M, Bradshaw CS, Cone RA (2017). The role of lactic acid production by probiotic Lactobacillus species in vaginal health. Res. Microbiol..

[17] Ravel J (2011). Vaginal microbiome of reproductive-age women. PNAS..

[18] European Food Safety Authority. Introduction of a Qualified Presumption of Safety (QPS) approach for assessment of selected microorganisms reffered to EFSA- Opinion of the scientific committee. 77 (2007).

[19] Kankainen, M. *et al*. Comparative genomic analysis of Lactobacillus rhamnosus GG reveals pili containing a human-mucus binding protein. *Proc. Natl. Acad. Sci. USA* **106** (2009). 10.1073/pnas.0908876106 PMC2746127 19805152

[20] Kleerebezem M (2003). Complete genome sequence of Lactobacillus plantarum WCFS1. PNAS..

[21] Anukam KC (2013). Genome Sequence of Lactobacillus pentosus KCA1: Vaginal Isolate from a Healthy Premenopausal Woman. PLoS One.

[22] Segers ME, Lebeer S (2014). Towards a better understanding of Lactobacillus rhamnosus GG - host interactions. Microb. Cell Fact..

[23] van den Nieuwboer M, van Hemert S, Claassen E, de Vos WM (2016). Lactobacillus plantarum WCFS1 and its host interaction: a dozen years after the genome. Microbial Biotechnology.

[24] Tapiovaara L (2016). Absence of adverse events in healthy individuals using probiotics - analysis of six randomised studies by one study group. Benef. Microbes.

[25] van Baarlen, P. *et al*. Differential NF-kB pathways induction by Lactobacillus plantarum in the duodenum of healthy humans correlating with immune tolerance. *Proc. Natl. Acad. Sci*., 10.1073/pnas.0809919106 (2009). 10.1073/pnas.0809919106 PMC2650163 19190178

[26] van Baarlen P (2011). Human mucosal *in vivo* transcriptome responses to three lactobacilli indicate how probiotics may modulate human cellular pathways. PNAS..

[27] Skovbjerg S (2009). Spray bacteriotherapy decreases middle ear fluid in children with secretory otitis media. Arch. Dis. Child..

[28] Reid G, Bruce AW (2001). Selection of Lactobacillus Strains for Urogenital Probiotic Applications. J. Infect. Dis..

[29] Aleshkin VA, Voropaeva EA, Shenderov BA (2006). Vaginal microbiota in healthy women and patients with bacterial vaginosis and nonspecific vaginitis. Microb. Ecol. Health Dis..

[30] Yoon SH (2017). Introducing EzBioCloud: A taxonomically united database of 16S rRNA gene sequences and whole-genome assemblies. Int. J. Syst. Evol. Microbiol..

[31] Chew SY, Cheah YK, Seow HF, Sandai D, Than LTL (2015). Probiotic Lactobacillus rhamnosus GR-1 and Lactobacillus reuteri RC-14 exhibit strong antifungal effects against vulvovaginal candidiasis-causing Candida glabrata isolates. J. Appl. Microbiol..

[32] van den Broek MFL, De Boeck I, Claes IJJ, Nizet V, Lebeer S (2018). Multifactorial inhibition of lactobacilli against the respiratory tract pathogen Moraxella catarrhalis. Benef. Microbes.

[33] Allonsius CN (2017). Interplay between Lactobacillus rhamnosus GG and Candida and the involvement of exopolysaccharides. Microb. Biotechnol..

[34] Allonsius CN (2019). Inhibition of Candida albicans morphogenesis by chitinase from selected lactobacilli. Sci. Rep..

[35] Malik S (2013). The highly autoaggregative and adhesive phenotype of the vaginal Lactobacillus plantarum strain CMPG5300 is sortase dependent. Appl. Environ. Microbiol..

[36] Donders GGG, Vereecken A, Bosmans E (2002). Definition of a type of abnormal vaginal flora that is distinct from bacterial vaginosis: aerobic vaginitis. Brit. J. Obs. Gynaec..

[37] De Boeck I (2017). Comparing the healthy nose and nasopharynx microbiota reveals continuity as well as niche-specificity. Front. Microbiol..

[38] Op De Beeck, M. et al. Comparison and validation of some ITS primer pairs useful for fungal metabarcoding studies. PLoS One 9, (2014). 10.1371/journal.pone.0097629 PMC4059633 24933453

[39] Kozich JJ, Westcott SL, Baxter NT, Highlander SK, Schloss PD (2013). Development of a dual-index sequencing strategy and curation pipeline for analyzing amplicon sequence data on the miseq illumina sequencing platform. Appl. Environ. Microbiol..

[40] Callahan BJ (2016). DADA2: High-resolution sample inference from Illumina amplicon data. Nat. Methods.

[41] Kõljalg U (2005). UNITE: a database providing web-based methods for the molecular identification of ectomycorrhizal fungi. New Phytol..

[42] Jervis-Bardy J (2015). Deriving accurate microbiota profiles from human samples with low bacterial content through post-sequencing processing of Illumina MiSeq data. Microbiome.

[43] Lebeer, S. *et al*. Topical cream with live lactobacilli modulates the skin microbiome and reduce acne symptoms. bioRxiv 463307, 10.1101/463307 (2018).

[44] Martin, R. *et al*. The Candida albicans-specific gene EED1 encodes a key regulator of hyphal extension. *Plos One* **6** (2011). 10.1371/journal.pone.0018394 PMC3075580 21512583

[45] Danielsen M (2002). Characterization of the tetracycline resistance plasmid pMD5057 from Lactobacillus plantarum 5057 reveals a composite structure. Plasmid.

[46] Malik, S. et al. Draft genome sequence of Lactobacillus plantarum CMPG5300, a human vaginal isolate. *Genome Announc*. **2** (2014). 10.1128/genomeA.01149-14 PMC4241660 25395634

[47] Chan RC, Bruce AW, Reid G (1984). Adherence of cervical, vaginal and distal urethral normal microbial flora to human uroepithelial cells and the inhibition of adherence of gram-negative uropathogens by competitive exclusion. J. Urol..

[48] Chan RCY, Reid G, Irvin RT, Bruce AW, Costerton JW (1985). Competitive exclusion of uropathogens from human uroepithelial cells by Lactobacillus whole cells and cell wall fragments. Infect. Immun..

[49] Reid G, Reid G (1999). The Scientific Basis for Probiotic Strains of Lactobacillus. Appl. Envir. Microbiol..

