# Supplementary material for: Impact of a lactobacilli-containing gel on vulvovaginal candidosis and the vaginal microbiome
Source: Sci Rep. 2020 May 14;10:7976. doi: 10.1038/s41598-020-64705-x (PMC7224289; doi:10.1038/s41598-020-64705-x)
Supplement: Supplementary file 1 — Supplementary information. [file 41598_2020_64705_MOESM1_ESM.pdf]

Supplementary information for:

Impact of a lactobacilli-containing gel on vulvovaginal candidosis and the vaginal microbiome

Eline F.M. Oerlemans, Gert Bellen, Ingmar Claes, Tim Henkens, Camille Nina Allonsius, Stijn Wittouck,

Marianne F.L. van den Broek, Sander Wuyts, Filip Kiekens, Gilbert G.G. Donders, Sarah Lebeer

Supplementary tables

Supplementary table S1: Inclusion and exclusion criteria applied in the proof-of-concept study

Inclusion and exclusion criteria:
• Informed consent must be signed
• 18-50 years of age
• VVC diagnosis: Positive Candida microscopy and/or culture
• Positive for 2 or more of following symptoms: vulvovaginal burning, (postcoital) itching, redness, fissures/excoriations, discharge, vulvar edema, and/or postcoital lesions (scored as absent (0), mild (1), moderate (2) or severe (3)).
• No use of vaginal products, oral antimycotic and/or antibiotics from one week prior until the end of the study
• No pregnant or lactating women
• No women not using contraception
• No vaginal douching (less than 48h prior to study)

Supplementary table S2: Some important findings from the questionnaires taken at visit 1 and visit 4.

Questionnaires	Rescue medication received<br>N=11	No rescue medication received<br>N=9
Age	42.4 +/- 9.9	35.47 +/- 12.64
Number of previous *Candida* infections	6.00 +/- 6.29	3.33+/- 5.24
Women with recurrent VVC (*1)	N=11	N=5
To use in future (*2) :<br>unlikely<br>Possibly<br>probably	 5 (50%)<br>3 (30%)<br>2(20%)	 0<br>3 (33%)<br>6 (67%)

(*) p=0,023

(*2) data of one questionnaire is missing (of the rescue medication group)

Supplementary table S3: Inventory of primer sequences of primers used in this study

Target gene	***Primer***	***Sequence***	***Source***
PPIA	PPIA_F	GCT TGC TGG CAG TTA GAT GTC	Jacobsen *et al.* (2014) [46]
	PPIA_R	AGA GGT CTG TTA AGG TGG GC	
GAPDH	GAPDH_F	ATT TGG CTA CAG CAA CAG GG	Jacobsen *et al.* (2014) [46]
	GAPDH_R	TCA AGG GGT CTA CAT GGC A	
Interleukin 8	IL8_F	TGG CAG CTT TCC TGA TTT CT	Moretti *et al.* (2019) [47]
	IL8_R	TTA GCA CTC CTT GGC AAA CT	
ITS	SA501_ITS86F	AAT GAT ACG GCG ACC ACC GAG ATC TAC ACA TCG TAC GGC CGG TCG AGT AGT GAA TCA TCG AAT CTT TGA A	ITS86F [48] and ITS4 [49] adapted as described in Kozich *et al.* (2013) [50]
	SA701_ITS4	CAA GCA GAA GAC GGC ATA CGA GAT AAC TCT CGA GTC AGT CAG GGT CCT CCG CTT ATT GAT ATG C	
V4 region of 16s rRNA gene	V4.SA701	CAA GCA GAA GAC GGC ATA CGA GAT AAC TCT CGA GTC AGT CAG CCG GAC TAC HVG GGT WTC TAA T	Kozich *et al.* (2013) [50]
	V4.SA501	AAT GAT ACG GCG ACC ACC GAG ATC TAC ACA TCG TAC GTA TGG TAA TTG TGT GCC AGC MGC CGC GGT AA	

Supplementary figures

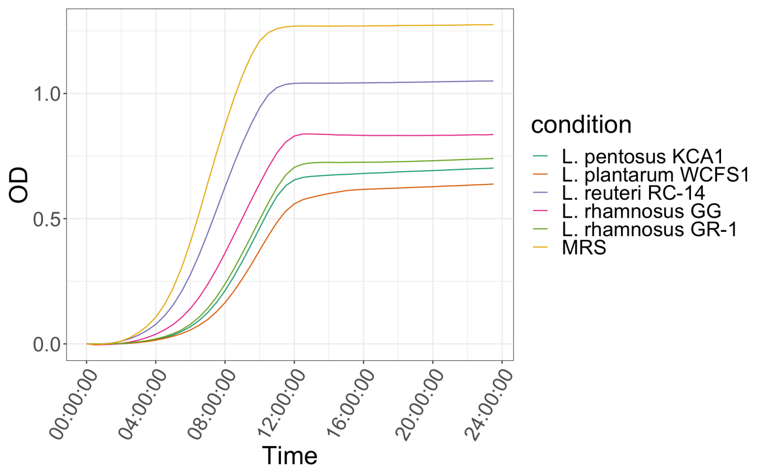

Supplementary figure S1: Time course analysis of spent culture supernatant on *C. albicans* growth. Time-course analysis was performed as described in Van Den Broek et al. 2018 [43] with minor adjustments. CFS of the lactobacilli was produced as described in Materials and Methods and added to YPD medium at a concentration of 20% v/v. This was inoculated with an overnight culture of *C. albicans* at 1% (circa 10^4 CFU/ml). A negative control was included, more precisely 20% MRS medium at pH4. Growth was evaluated by optical density at 600nm, using Synergy HTX multi-mode-meter (Biotek).

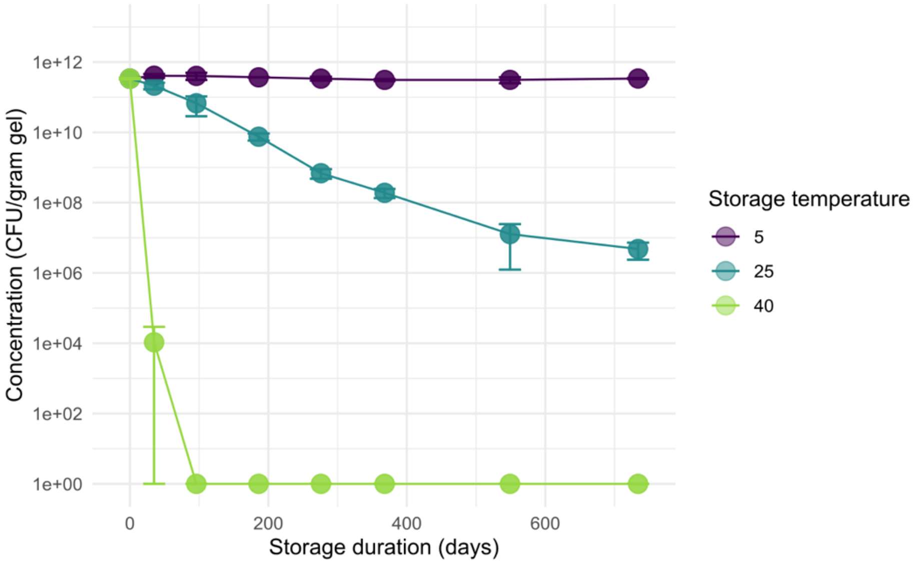

Supplementary Figure S2: Stability testing of the probiotic gel according to ICH Q1A (R2) guideline [30]. The viability of lactobacilli in the silicone gel was evaluated after storage at 5°C, 25°C and 40°C, over a two-year period. Three tubes were tested at each time point and the concentration of viable lactobacilli was estimated by plating, performed in triplicate for each tube.

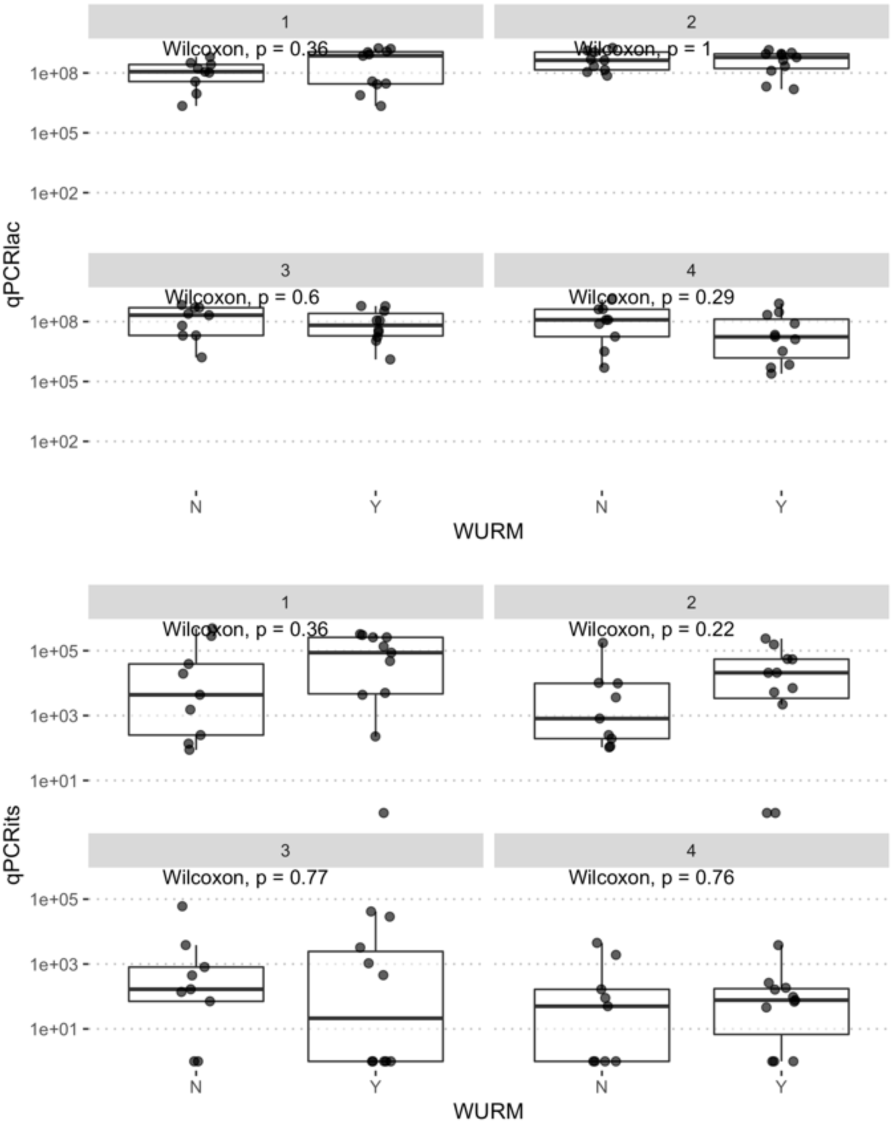

Supplementary figure S3: Differences between women that did (Y) or did not (N) use rescue medication, in estimated absolute abundances for lactobacilli (a) and fungi (b), facetted by visit number.

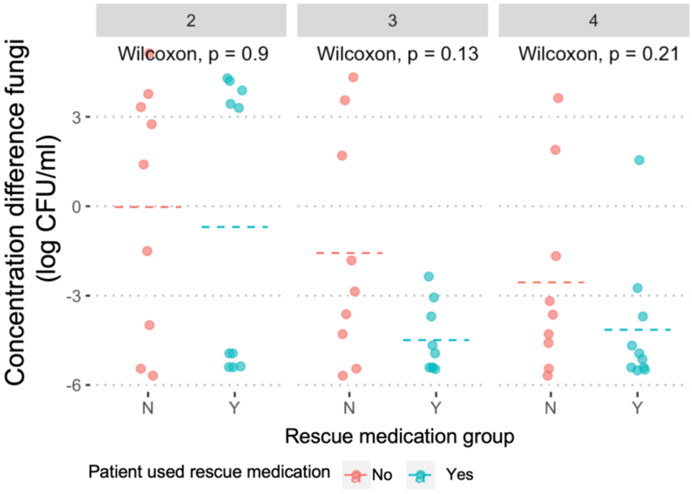

Supplementary figure S4: Differences in concentration of fungi over the course of the study as compared to study onset (visit 1). Concentration differences were calculated, and log transformed.

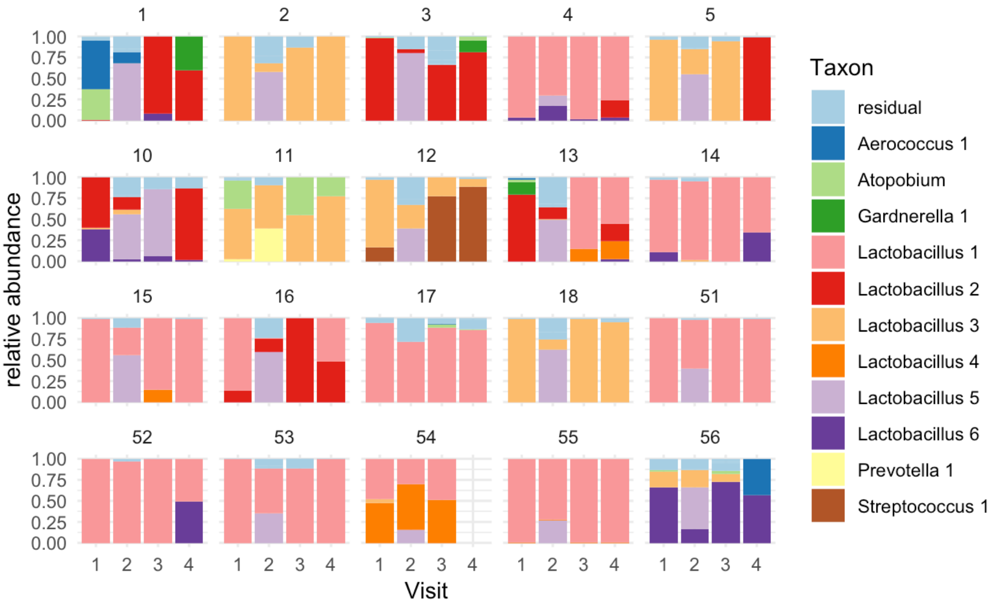

Supplementary figure S5: Bacterial community (*16S rRNA* sequencing profile) of samples of proof-of-concept study with vaginal gel containing lactobacilli to combat VVC. Samples are ordered by patient (indicated at the top of each graph and by visit (x-axis). Relative abundance (y-axis) is shown for the top 11 ASVs (indicated by color), while the remaining ASVs are grouped as ‘residual’ (light blue).

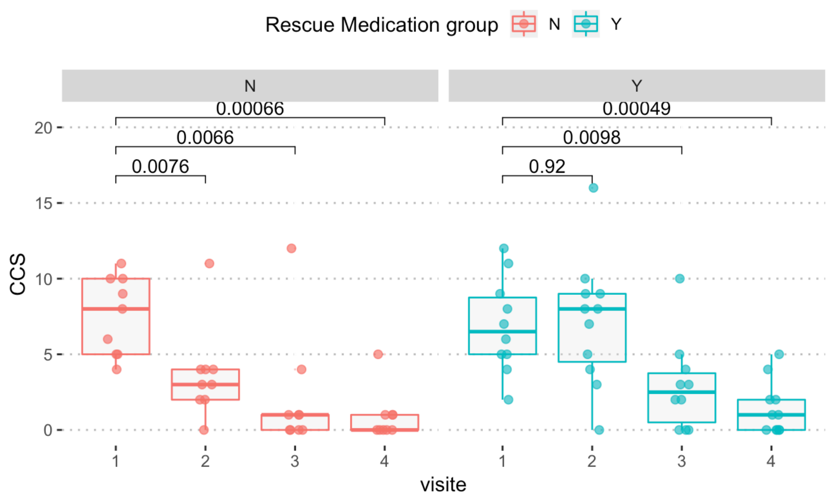

Supplementary figure S6: Evolution of the clinical composite score (CCS) of the patients during the proof-of-concept study. This CCS takes into account the following symptoms, scored as absent (0), mild (1), moderate (2) or severe (3): vulvovaginal itching, burning, redness, fissures and edema.

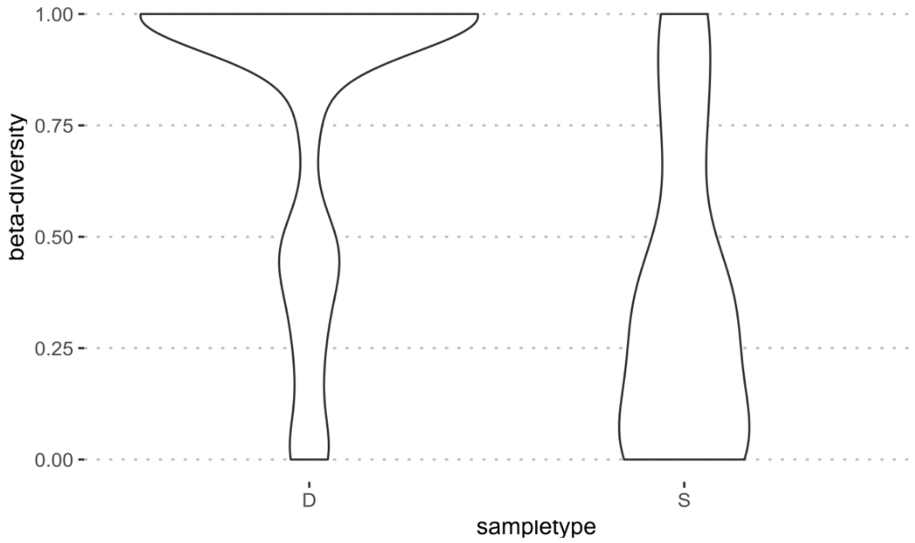

Supplementary figure S7: Beta-diversity of *16S rRNA* samples as Bray-Curtis dissimilarity index between samples of visit 1 and visit4 of samples of the same participant (S) or different participants (D). A low beta -diversity indicates samples are similar, while a high beta-diversity index indicates samples are very different.

Supplementary materials and methods

Spot assay

Overnight cultures of lactobacilli were spotted (2µl) onto an MRS base. A negative and positive control of sterile water and 0.2% miconazole was included. After 48 hours of growth, YPD soft agar containing 0.2% v/v of an overnight *Candida albicans* culture (circa 2.10^3 CFU/ml) was poured over the MRS base. Following 18h of incubation at 37°C, the radius of the spots and inhibition zones were measured. The width of the halo was calculated by subtracting the radius of the spot from the radius of the inhibition zone.

Hyphae

Fetal bovine serum (Perbio Science) was supplemented in YPD broth to induce hyphae in *C. albicans* (10^6 CFU ml $^{-1}$), in pure culture or in co-incubation lactobacilli (10^8 CFU ml $^{-1}$). After incubation, the ratio of hyphae to yeast cells was calculated from at least 100 yeast cells and/or hyphae in at least three biological repeats.

Adhesion and adhesion competition assays

Lactobacilli were grown overnight, spun down (10 min, 4000g), washed in phosphate buffered saline (PBS) and resuspended in the VK2/E6E7 cell medium to an estimated concentration of 1.10^8 or 2.10^8 CFU ml^{-1} for the adhesion and the adhesion-competition assays respectively. For the latter experiment, overnight *C. albicans* cultures, were treated similarly and resuspended to a concentration of 2.10^6 cells ml^{-1}. For the adhesion assay of the lactobacilli, 1ml of one of the suspensions of lactobacilli (1.10^8 CFU ml^{-1}) was applied to a monolayer of VK2/E6E7 cells. To test adhesion-competition, 0.5ml of suspension of one of the lactobacilli was added to the cells immediately followed by 0.5ml of the *C. albicans* suspensions. After 2 hours of co-incubation, the suspension was removed, the cells were washed twice with PBS, detached using 200µl of trypsin (0.25%). The bacterial/ fungal concentrations of this suspension and the original suspensions were estimated using serial dilutions and plating on MRS and Sabouraud solid media (Carl Roth) for lactobacilli and *C. albicans* respectively.
